# PROTOCOL: Strategies for scaling up the implementation of interventions in social welfare: protocol for a systematic review

**DOI:** 10.1002/CL2.201

**Published:** 2018-09-24

**Authors:** Luke Wolfenden, Bianca Albers, Aron Shlonsky

## 1. Background

### 1.1 The problem, condition or issue

Social welfare organisations provide care, support and protection to children or adults at risk of, or with needs arising from, mental illness, disability, age and poverty.

There is increasing social welfare sector interest in integrating effective practices into the array of services that are provided to citizens by both public and private provider agencies (Montero, 2015; Supplee & Metz, 2014). Yet maximising the benefits of effective interventions addressing the needs of children, adults and families in adversity requires these interventions to be implemented at scale, providing high quality interventions to large numbers of individuals who require various types of support.

Social welfare interventions, even if found to have a treatment effect, can fail due to poor implementation. That is, recipients of a potentially effective service can only benefit from a service they actually receive. However, the implementation of potentially effective interventions in social welfare is still sporadic, localised, and can have considerable geographic variation (Walker et al., 2016). There are numerous reasons for uneven uptake. Barriers in social service organisations and their workforce may hamper the systematic uptake of effective practices due to a lack of training and support (McBeath et al., 2015; McBeath & Austin, 2014). Existing attitudes towards and habits for utilising research in social work practice (Wutzke et al., 2016; Kreisberg & Marsh, 2016; Knight, 2013; Smith, 2013) may have the same effect due to the general complexity that characterises processes of evidence integration in routine service settings (Carnochan et al., 2017).

There is also evidence to suggest that high quality implementation of even modestly effective programs can result in better outcomes than the poor implementation of programs that have been found to be highly effective (Lipsey, 2009; Lipsey, Howell, Kelly, Chapman & Carvin, 2010). Understanding how interventions can be scaled up from single trials and local innovation projects to fully implemented practices and programs that reach their intended entire population is a priority within social welfare.

### 1.2 The intervention

‘Scaling up’ or the concept of ‘scalability’ has been variously defined in the literature.

Perhaps the most widely used definition of ‘scaling up’ is that provided by the World Health Organization (WHO) in 2009 and since adopted by other agencies within health and human services including the recently published European Scaling Up Strategy in Active and Healthy Ageing (European Commission, 2015). Specifically, WHO defines ‘scaling up’ as “deliberate efforts to increase the impact of health service innovations successfully tested in pilot or experimental projects to benefit more people and to foster policy and programme development on a lasting basis” (WHO, 2009: 1).

Scalability is defined as the the ability of a health intervention shown to be efficacious on a small scale and / or under controlled conditions to be expanded under real world conditions to reach a greater proportion of the eligible population, while retaining effectiveness (Milat et al., 2012). At its core, the concept includes an explicit intent to expand the reach of an intervention. Implementation – “the use of strategies to adopt and integrate evidence‐based health interventions and to change practice patterns within specific settings” (Glasgow et al., 2012, p.1275) – is a key feature of the process of scaling up as sufficient implementation is necessary for scaled interventions to be beneficial. As such, all implementation strategies could be considered relevant to scaling interventions. The Expert Recommendation for Implementing Change Project, for example lists 73 implementation strategies including providing technical assistance using an implementation advisor, and mandating change (Powell et al., 2015). However, the process of scaling interventions includes consideration of a range of pre‐conditions and strategies that typically precede implementation efforts, including constituency building and realigning resources and infrastructure to enable delivery at scale (Milat et al., 2015).

There have been a number of examples of attempts to scale‐up implementation of social welfare interventions.

The Parent Management Training Oregon Model is a curriculum‐based training program for parents that seeks to reduce or prevent child externalising behaviour problems and is typically provided over 25 one‐hour sessions by trained therapists. Following positive findings from two randomised trials, PMTO was scaled up across Norway. Implementation was supported by investment in a national centre to establish implementation infrastructure. Implementation support strategies included therapist training, the establishment of regional therapist networks, on‐site coaching, monitoring, accreditation processes and technical support. Evaluation of the initiative suggests that the program fidelity was maintained, and effects of the program were comparable with those reporting in efficacy trials (Tommeraas et al., 2017).

Brown and colleagues (2014) report a randomised trial of comparing two different approaches to the implementation of Multidimensional Treatment Foster Care ‐ an evidence‐based intervention involving the placement of children in supported community‐based foster care rather than aggregated care settings. Implementation strategies delivered to one group included technical support, face to face stakeholder meetings, the development of action plans, training of administrators, supervisors and therapists and parents, and on‐going consultation. A comparison group received the same implementation support strategies but also received additional technical assistance from program consultants during peer to peer meetings and monthly conference calls and facilitating communities of practice (quality improvement collaboratives). The trial identified little difference in implementation between groups at follow‐up (Brown et al., 2014).

Several frameworks have been published describing approaches to scale interventions. Such frameworks demonstrate that interventions to implement innovations at scale are complex, and involve the consideration of a number of individual, organisational, social, political and other contextual factors (Barker et al., 2016; Milat et al., 2016; Seay et al., 2015; Atkinson et al., 2013; Kohl & Cooley, 2003). Interventions to achieve implementation at scale, therefore, are not uniform, and are likely most effective if they consider and address factors that impede achievement of large scale implementation.

### 1.3 How the intervention might work

While some already consider the small‐scale transport of effective interventions into real life settings (e.g., through a single project or trial site) as a part of scaling up effective practice (Dunlap et al., 2009), this review will focus on system‐, state‐, nation‐based or global scale up efforts aiming to integrate effective interventions within social welfare to entire relevant populations.

Implementation science is the study of methods and strategies to promote the uptake of interventions that have proven effective into routine practice, with the aim of improving population health^1^. ‘Scaling‐up’ is a broadly acknowledged concept within implementation science (Dymnicki et al., 2017; Hoagwood et al., 2014; Milat et al., 2012; Norton & Mittman, 2010), and several strategies have been suggested as effective by different researchers based on multiple studies. Among scale up strategies mirrored in this literature are e.g., ‘research‐practice collaborations’ (Chamberlain et al., 2012), advocacy and stakeholder activation; resource allocation; capacity building (Resnick and Rosenheck, 2009; Hurlburt et al., 2014); system restructuring (Eaton et al., 2011), use of business practices and technologies; quality assessment and evaluation (Hoagwood et al., 2014); interagency collaboration (Aarons et al., 2014; Hurlburt et al., 2014) and the adaptation and maximisation of fit between intervention and context (Klingner, Boardman & Macmaster, 2013).

However, a set of commonly agreed upon strategies or actions necessary to include in effective scaling efforts has not emerged from this literature. Nonetheless, frameworks consolidating behavioural and organisational theories that have been applied to improve the scale of implementation of interventions have identified a number of factors that can impede or facilitate scaling. For example, a recent review of frameworks (Atkinson et al., 2013) identified a range of factors that need to be in place prior to larger scale roll‐out of interventions including leadership structures, infrastructure, and alignment between intervention goals and organisational priorities. During the implementation phase, however, facilitators include attention to changing organisational culture., equipping frontline staff with tools for problem solving, and implementation monitoring (ibid.). While the mechanisms of effects of scale‐up strategies differ based on the contexts and determinants of implementation, best practice approaches recommend formative evaluation to identify scale‐up barriers and developing strategies to address them, thereby enhancing the likeliness of increasing their impact.

### 1.4 Why it is important to do the review

The heterogeneity mirrored by just these few articles on questions of scale up, highlights that it is difficult at the current developmental stage of implementation science to – a priori – identify a standardised set of individual, organisational and system strategies that researchers have agreed upon as necessary to include in any type of scaling attempt. A systematic review aiming to synthesise the current best evidence on the effectiveness of strategies to scale up potentially effective social welfare interventions therefore can help inform both practice and research environments with a strong focus on the implementation of effective services.

A small number of systematic reviews address implementation in general within health and education without addressing particular questions of scaling (Greenhalgh et al., 2004; Francke et al., 2008; Chaudoir et al., 2013; Gibson et al., 2015; Naylor et al., 2015). Fewer still specifically focus on scale up.

In examining the literature on effective dissemination and implementation interventions to support cancer prevention, Rabin et al. (2010) point to the high level of heterogeneity in language, study population & design, measures and other study characteristics, making it difficult to draw firm conclusions about effective strategies. Through a rapid review, Atkinson et al. (2013) succeeded in identifying 21 frameworks for facilitating large‐scale changes in health services. Based on the seven of these that had been applied in quality improvement initiatives, the authors derived 13 key factors important to large scale change.

Milat et al. (2015) use a similar approach in conducting a narrative review of eight frameworks for scaling health interventions. They point to several common cross‐framework characteristics, one of which is the requirement to have a well‐defined scale up strategy. However, the steps involved in developing such a strategy are again highly different from framework to framework.

With a focus on the UK, Pearson et al. (2015) examine the conditions and actions supporting the implementation of health promotion programmes in schools. They conclude that while the literature provides insights into some aspects of programme implementation, the knowledge about several implementation practices remains underdeveloped, among others because studies often do not reach the scaling stage of implementation due to limitations put on funding.

Finally, strategies to effectively implement policies and practices to improve child health through childcare services were the focus of a systematic review by Wolfenden et al. (2015). Like other publications, the authors point to weak and inconsistent evidence in this area, making it impossible to point to particularly effective strategies for high quality large scale implementation.

Similarly, very few systematic reviews in social welfare focus on dissemination and/or implementation in general (Novins et al., 2013; Leeman et al. 2015).

We are not aware of any systematic review, completed or in progress, that specifically focuses on scale up in a social welfare context. Further, social welfare agencies have specific values, governance structures, resources, workforce capacity and cultures that are different from those present in health services. As such, contextual factors are important considerations in the process of scaling effective interventions (Milat et al., 2015), and the findings of existing reviews may not generalise to the social welfare sector.

Given this lack of clarity around scaling terminology and the scarcity of knowledge about effective scaling, this systematic review has the potential to contribute to:

- Building the evidence base of scaling and informing policy and practice about effective approaches to scaling potentially effective interventions.

## 2. Objectives

The primary objective of this review is to assess the effectiveness of strategies aiming to support the scale‐up of interventions in social welfare.

Hence, the research question guiding this systematic review is:

***Among social welfare services, how effective are strategies seeking to improve the implementation of effective interventions, programs or services at scale?***

Additionally, the review seeks to describe the context in which implementation at scale occurs.

## 3. Methodology

### 3.1 Criteria for including and excluding studies

#### 3.1.1 Types of study designs

Given the potentially complex nature of evaluation studies of implementation trials, we will include a broad range of study designs in this review.

While randomized controlled trials (RCTs) are the most internally valid design to assess the effectiveness of a scaling strategy, such designs may not always be the most appropriate research designs for evaluating the impact of such strategies. For example, there may be too few allocation units available for baseline equivalence when measuring scaling strategies that target large geographic regions, such as provinces, counties, states or nations. Furthermore, given that implementation science (more broadly) and scale up (more specifically) are relatively new fields of science, large numbers of randomised trials are unlikely, and the inclusion of non‐randomised trials may increase the pool of available studies, providing a more comprehensive evidence‐base for policy and practice decision‐making. We therefore will include any study that uses one of the following designs:

- RCTs and cluster RCTs
- Quasi‐RCTs and cluster quasi‐RCTs (e.g., step‐wedge)
- Controlled before‐and‐after studies (CBAs) and cluster CBAs
- Time series research designs
- Regression discontinuity designs
- Difference of difference or other econometric designs
- Propensity score matching and other matching designs

Qualitative and other uncontrolled observational studies may be useful for understanding why strategies succeed or fail, but this review is focused on establishing whether strategies are effective. As such, we will only include quantitative studies that

1. compare a strategy to achieve implementation of an intervention at scale with no strategy or ‘usual practice’,
2. compare two or more strategies to achieve implementation at scale, or
3. assess a single scaling strategy (given an appropriate design).

#### 3.1.2 Types of participants

Participants could include any social welfare organisation that provides care, support and protection services to children, adults, families and communities that are at risk of or already require support due to adversities arising from mental illness, disability, age or poverty.

This includes social welfare organisations operating in the areas of child welfare and child protection; mental health and substance abuse; juvenile justice; housing; aged care and employment.

Included are both the internal stakeholders to social welfare organisations ‐ their staff, clients, and administrators ‐ and their external stakeholders responsible for e.g. their financing, regulation and development. This group includes representatives for e.g. government bodies, regulatory agencies or for intermediaries that have a capacity to provide service agencies with professional supports and technical assistance.

Studies in which only a subset of the sample is eligible for inclusion – e.g. if a study covers both social welfare and health organisations – will be excluded.

#### 3.1.3 Types of interventions

A number of reviews have been conducted in hospitals and primary care settings to assess strategies to scale evidence‐based health services. However, operating contexts, processes and structures of medical settings differ considerably from social welfare organisations, and as such, the effects of implementation approaches may not generalise across these settings.

This systematic review, therefore, focuses on strategies aiming to scale up the implementation of discrete, potentially effective social welfare interventions in federal, state, community and individual settings including social assistance offices, community based mental health clinics, neighbourhood initiatives, individual households, and individuals within households. Studies of strategies to scale interventions in medical settings such as hospitals or general practice will be excluded, as will those in educational settings such as schools or universities.

We will include trials of any strategy that seeks to increase the scale of implementation of social welfare interventions. A range of potential strategies could be used to improve implementation of social welfare interventions at scale. (Powell et al., 2012 & 2015).

For example, the Cochrane Effective Practice and Organisation of Care Taxonomy (EPOC, 2016) lists strategies pertaining to delivery arrangements (e.g. co‐ordination of different providers), financial arrangements (e.g. pay for performance – targeted payments), governance arrangements (e.g. decentralisation of authority for health services) and implementation strategies (e.g. audit and feedback). Implementation strategies have also been characterised by the Expert Recommendation for Implementing Change (ERIC) Project, and scale‐up frameworks suggest a range of strategies that can be enacted, before during and following the implementation phases of the scale‐up process. We will include both individual strategies and combinations of strategies.

Additionally, to be included, studies are required to:

- Describe an initiative that sought to enhance the scale of implementation of a discrete social welfare intervention, program or service above the scale at which it had previously been implemented. For example, increasing scale could be achieved by supporting implementation of an intervention by a greater number of community organisations, or expanding the availability of social welfare sources.
- Seek to increase the scale of implementation of a potentially effective social welfare intervention, program or service.

Potentially effective programs of services will be defined as those that have been evaluated in at least one randomised controlled trial where at least one primary trial outcome had a statistically significant (P<0.05), positive effect favouring the intervention and no substantial adverse effects of the intervention (i.e., not the scale up) were reported. Randomised designs were selected as they provide good evidence to support effectiveness in evidence hierarchies (Evans, 2003). If the intervention, service or program has been subject to repeated assessment using randomised designs, pooled effect estimates on the primary trial outcome of the social welfare intervention must represent a significant improvement on the primary trial outcome (p>.05) relative to control or comparison group. Or ‐ if the pooled estimate was unavailable or impossible to be calculated ‐ the findings of the trial judged to represent the most valid estimate of effect will be used, based on consensus of two review authors and using the Cochrane Risk of Bias assessment tool.

#### 3.1.4 Types of outcome measures

To be eligible, trials must include a measure of implementation fidelity – the primary outcome of this review.

From such studies, data pertaining to secondary trial outcomes will also be assessed. We will include trials reporting post intervention follow‐up data only if baseline values can be assumed to be zero, as would be the case for trials attempting to scale up implementation of an intervention, program or service that was not available prior to study commencement, or if the trial employed a randomised design, where by baseline equivalence can be assumed (or differ only due to chance).

There will be no exclusion criteria on the source of outcome data. Data for the primary and secondary outcome measures can be obtained from any course including institutional records, direct observations, surveys or questionnaires completed by social welfare organisation staff or clients.

The included primary and secondary outcome measures and their operational definitions have been based on the heuristic and working taxonomy of outcomes proposed by Proctor et al. (2011). The included measures are also inclusive of factors recommended in different evaluation frameworks, including the RE‐AIM (Reach Effectiveness Adoption Implementation Maintenance) framework (Glasgow et al., 2009; Gaglio et al., 2013).

**Primary outcomes:**

1. ***Implementation***: Any measure of the fidelity of social welfare interventions, programs or service implementation will be included. Fidelity is defined as the degree to which an intervention was implemented as it was intended. Measures could include level of adherence with a protocol, dose or number of elements implemented, or measures of quality of delivery.

**Secondary outcomes:**

1. ***Adoption*** – For adoption, we will include any measure of uptake, including an intention, initial decision, or action to try and implement potentially effective social welfare interventions, programs or services. These could include decisions by managers of social welfare organisations to take‐up a potentially effective service, or individual staff intentions to deliver potentially effective services to clients.
2. ***Penetration****–* Penetration is the integration of a practice within a service setting or its sub‐settings. It is comparable to conceptual definitions of intervention ‘reach’. We will include any measure of penetration at the individual client, or organisational level, for example, the proportion of eligible individuals (or organisations) that receive an intervention (or implement an intervention) of the total number eligible to do so. These could include the proportion of all clients of a community service organisation eligible for a service that actually receive it.
3. ***Sustainability of implementation***: We will define sustainability as the extent to which a newly implemented intervention, program or service is maintained. Measures of sustainability, therefore, must first require successful implementation in part or in full, of an intervention, program or service. We will include any measure of ongoing sustainability of implementation of intervention elements assessed at least 6 months following a measure of successful implementation. This could include e.g. the proportion of community service organisations maintaining implementation of all elements of a program 12 months following provision of implementation support.
4. ***Effectiveness*** – We will include measures of the effectiveness of the social welfare intervention implemented and scaled based on the disease, condition, state or circumstance that it was intended to improve. These could include measures of health status, disability, educational status or other behaviours of clients of social welfare services.
5. ***Acceptability*** – We will include any measure of the acceptability, defined as the perception among implementation stakeholders that a given treatment service, practice or innovation is agreeable, palatable or satisfactory. Measures of acceptability assessed at the individual or organisational level will be included such as surveys of staff or managers of social welfare organisation regarding their experience of features of the intervention (e.g. scheduling, or office environment).
6. ***Appropriateness*** – we will include any measure of the appropriateness, defined as the perceived fit, relevance or compatibility of an innovation of evidence‐based practice for a given setting, provider or consumer, and/or perceived fit of the innovation to address a particular problem. Measures of appropriateness assessed at the individual or organisational level will be included such as surveys of staff or managers of social welfare organisation regarding their perception of the consistency of the implementation of a new intervention with their skill set, role or work expectations.
7. ***Feasibility*** – Feasibility is defined as the extent to which a new treatment, or an innovation can be successfully used or carried out within a given agency or setting. We will include only measures of resource, infrastructure or cost aspects of the feasibility of delivery of the intervention such as the existence of tools required for intervention implementation. Measures of feasibility assessed at the individual or organisational level will be included
8. ***Costs*** –We will include any reports of any measures of cost (absolute, incremental, cost ratios) associated with the impact of an implementation effort, including costs of the intervention program or service, or the implementation strategy. Costs could relate to new facilities, the expansion of training, hiring new staff, communication activities, or licenses.

#### 3.1.5 Adverse effects

We will include any measure of unintended adverse effects from strategies to increase the scale of implementation of potentially effective social welfare interventions for either individuals, or social welfare organisations. These could include adverse changes to the moral of staff or their working conditions, displacement or defunding of other potentially effective interventions with greater empirical support or worsening of conditions of service recipients due to poor, incorrect or unsafe implementation practices. All adverse effects described in eligible studies will be included in the synthesis.

#### 3.1.7 Duration of follow‐up

There will be no restriction on the length of the study follow‐up period.

#### 3.1.8 Types of settings

This systematic review will focus on the scaling of social welfare interventions in federal, state, community and individual settings including social assistance offices, community based mental health clinics, neighbourhood initiatives, individual households, and individuals within households (see also 3.1.2).

Studies of strategies to scale interventions in medical settings such as hospitals or general practice will be excluded, as will those in educational settings such as schools or universities.

#### 3.1.9 Other eligibility criteria

There will be no restriction on the language of publication. We will identify suitably qualified translators for studies identified published in non‐English languages. We will include studies in the peer reviewed and grey literature.

Any changes in eligibility criteria will be agreed prospectively between the members of the review team. These will be documented and reported as a discrepancy from protocol in the manuscript. In the advent of a change in eligibility, we will re‐screen citations.

### 3.2 Search strategy

The following electronic databases will be searched: Medline, Embase, PsycInfo, Cochrane Central Register of Controlled Trials (CENTRAL), CINAHL, Education Resources Information Center (ERIC), International Bibliography of the Social Sciences, Applied Social Science Index and Abstracts (ASSIA), Sociological Abstracts, Social Services Abstracts, Web of Science incl. Social Sciences Citation Index and Conference Proceedings Citation Index‐ Social Science & Humanities, Campbell Collaboration, and Criminal Justice Abstracts.

Search terms will be developed based on terminology representative of implementation and dissemination research (Rabin, 2008) and include search filters used in previous reviews (Rabin 2010; Wolfenden 2014). The search strategy for Medline is presented in Appendix 1 and will be adapted for the other databases by an experienced librarian, who also will conduct the searches. There will be no date of publication or language restrictions.

Protocols for all included trials will be sourced. Reference lists of all included trials will be hand‐searched for citations of other potentially relevant trials. Previous reviews on the topic will be screened for relevant studies. Targeted hand searches of all publications in the journal *Implementation Science* will be conducted. Forward citation searches of included studies will be performed using Google Scholar.

Furthermore, we will conduct searches of the grey literature. We will develop a separate search strategy including all of the following elements:

1. **Searches of grey literature databases** such as ProQuest Dissertations and Theses; TROVE; Open Grey etc.
2. **Targeted Google searches** that will be based on the search terms used with electronic databases
3. The **targeted screening of relevant sector and implementation websites**. The table below includes examples of such websites. This list will be further expanded and refined as part of preparing the review work.
  **Table:** 

***Sector specific websites***	***Implementation specific websites***
Social Care Online: https://www.scie‐socialcareonline.org.uk/	Society for Implementation Research Collaboration: https://www.societyforimplementationresearchcollaboration.org/
Institute for Research and Innovation in Social Services (IRISS): https://www.iriss.org.uk/resources	The Consortium for Implementation Science: http://consortiumforis.org/
Social Policy and Practice: http://www.spandp.net/	The National Implementation Research Network: http://nirn.fpg.unc.edu/
PsycEXTRA: http://www.apa.org/pubs/databases/psycextra/	The European Implementation Collaborative: https://www.implementation.eu/
The What Works Centre for Children's Care: https://whatworks‐csc.org.uk/	The Centre for Effective Services: http://www.effectiveservices.org/
4. **Consultation with subject matter experts**. We will
  a. Invite our expert panel members (please refer to page 28‐30 for details) for direct input and information about publications focused on scaling in social welfare
  b. Request our expert panel to distribute a call for input and information about publications focused on scaling in social welfare to their networks
  c. Ask the following implementation science and practice networks to distribute the above call through their network distribution lists:
    i. The European Implementation Collaborative (EIC)
    ii. The Society for Implementation Research Collaboration (SIRC)
    iii. The Consortium for Implementation Science
    iv. The U.K. Implementation Society
    v. The Ireland Implementation Network
    vi. The Implementation Research Institute (IRI)
  d. Contact authors of included trials for information about any relevant ongoing or unpublished trial or grey literature publications they might be aware of.

We will document all steps of the search process in sufficient detail to ensure future replicability and correct reporting. This will include a PRISMA flowchart, registration of excluded studies and dates at which the search was conducted. If the initial search date is more than 12 months from the intended publication date, we will rerun searches and fully incorporate new eligible studies.

### 3.3 Description of methods used in primary research

A broad range of methods can be expected to be used as part of the studies to be identified for this review. They may include the collection of data through archives and administrative databases, surveys, standardised inventories and measures, documents, interviews etc.

### 3.4 Criteria for determination of independent findings

#### 3.4.1 Study selection

Two reviewers, who will not be blind to the author or journal information, will independently screen abstracts and titles. Screening of studies will be conducted using a standardised screening tool developed based on the resources for authors of systematic reviews available through the Cochrane EPOC group (EPOC, 2015), and will be piloted before use. The full texts of manuscripts will be obtained for all potentially eligible trials for further examination. For all manuscripts, information regarding the primary reason for exclusion will be recorded and documented in the excluded studies table. The remaining eligible trials will be included in the review. For these, any relevant retraction statements and errata will be examined for information.

Discrepancies between reviewers regarding study eligibility will be resolved by consensus. In instances where the study eligibility cannot be resolved via consensus, a third reviewer will make the final decision.

#### 3.4.2 Data extraction and management

Two review authors, also unblinded to author or journal information, will independently extract information from the included trials. This information will be recorded in a data‐extraction form that will be piloted before initiation of the review. Discrepancies between reviewers regarding data extraction will be resolved by consensus or if required via a third reviewer. One reviewer will transcribe information from data extraction forms into Rev Man meta‐analysis software and included study tables. Data transcription will be checked by a second reviewer. The following information will be extracted:

1. Study eligibility as well as the study design, date of publication, setting, country, participant (age, gender, ethnicity), service demographic, (service sector, size) socioeconomic characteristics (the education, income and ethnicity of service constituents), number of experimental conditions, as well as information to allow assessment of risk of study bias and assessment of the overall quality of evidence using the GRADE approach.
2. Characteristics of the intervention/ scale‐up strategy. We will extract data to describe the evidence‐based intervention being scaled‐up, including the intervention components, duration and modality of delivery as well as the effect size reported (and p‐value) of trials of its efficacy. We will extract data to describe the scale up strategy consistent with the recommendations of Proctor et al. (2013) to include data on the individual or organisation (the actor) who enacts the strategy (e.g. administrator, provider, advocate etc.), the action steps or processes that need to be enacted, the target of action (e.g. providers, organisations), when the strategy is used, and its dose. The theoretical underpinning of the intervention (if noted in the study) will also be extracted. We will use the Cochrane Effective Practice and Organisation of Care (EPOC) Taxonomy to classify implementation strategies.
3. Trial primary and secondary outcomes, including the data collection method, validity of measures used, effect size and measures of outcome variability’
4. Source(s) of research funding and potential conflicts of interest.

As part of data extraction, we will check the accuracy of all numeric data in the review. Where information is unavailable from published reports we will contact study authors to obtain such data.

Multiple reports of the same study will be collated to ensure that each study rather than each report is the unit of interest in the review.

### 3.5 Details of study coding categories

Risk of bias will be assessed independently by two reviewers using the risk of bias tool described in the Cochrane Handbook for Systematic Reviews of Interventions (Higgins & Green, 2011). The tool provides an overall risk of bias (‘high’, ‘low’ or ‘unclear’) assessment for each included study based on consideration of study methodological characteristics, random sequence generation, allocation concealment, protection against contamination, blinding of outcome assessment, baseline outcome, baseline characteristics, selective outcome reporting, missing outcome data and other risks of bias.

Judgements made will be justified with information included in studies or related documents. If these documents are not publicly available, this will be explicitly stated.

If required, a third reviewer will adjudicate discrepancies regarding the risk of bias that cannot be resolved via consensus.

An additional criterion ‘potential confounding’ will be included for the assessment of the risk of bias in non‐randomised trial designs (Higgins & Green 2011). Risk of bias for included studies will be documented in a ‘Risk of Bias’ table.

### 3.6 Statistical procedures and conventions

#### 3.6.1 Measures of treatment effect

In accordance with Campbell and Cochrane guidelines, we will try to maximise the likelihood to quantitatively synthesize studies. For the primary and secondary outcomes, attempts will be made to conduct meta‐analysis using data from included trials. For binary outcomes, the standard estimation of the risk ratio and a 95% confidence interval will be calculated. For continuous data, the mean difference will be calculated where a consistent measure of outcome is used in included trials.

Where different measures are used to examine the primary outcome, the standardised mean difference will be calculated where possible. Where data from the same outcome are reported, in some studies as dichotomous data and in others as continuous data, we will transform these to enable pooled estimates of effect if it is appropriate to do so.

Where outcome data are not presented in 2X2 tables or are not presented in means and standard deviations, we will attempt to transform available data into a usable effect size using the online calculator *Practical Meta‐Analysis Effect Size Calculator* (Wilson, n.d.).

If studies using different scales are combined, we will ensure that higher scores for continuous outcomes all have the same meaning for any particular outcome. Specifically, we will explain the direction of interpretation and report when reversing scores to align direction is done.

Finally, we will check continuous outcome measures for skewness and, if substantial departures from normality are observed, we will transform these data prior to meta‐analysis.

If we are unsuccessful at transforming the data, we will attempt to contact the author of the study and request additional data.

#### 3.6.2 Quality evidence assessment

The overall quality of evidence for each outcome will be rated using the GRADE system (Gyatt et al., 2011) by two reviewers, with any disagreements resolved via consensus, or if required by a third reviewer. The GRADE system defines the quality of the body of evidence for each review outcome, describing the extent to which one can be confident in the review findings. The GRADE system requires an assessment of methodological quality, directness of evidence, heterogeneity, and precision of effect estimates and risk of publication bias. GRADE quality ratings (from ‘very low’ to ‘high’) will be used to describe the quality of the body of evidence for each review outcome. All assessments of the quality of the body of evidence (e.g. downgrading or upgrading) will be justified and documented.

#### 3.6.3 Clustered studies

Clustered trials will be examined for unit of analysis errors. Trials with unit of analysis error will be identified in the risk of bias table. For cluster‐randomised trials that have performed analyses at a different level to that of allocation, without appropriate statistical adjustment for clustering, the trials' effective sample size will be calculated for use in meta‐analysis. The intra‐cluster correlation co‐efficient (intra‐cluster correlation) derived from the trial (if available), or from another source (for example using the intra‐cluster correlations derived from other, similar trials) will also be utilised. Calculation of the design effect will be performed using the formula provided in the Cochrane Handbook for Systematic Reviews of Interventions (Higgins & Green 2011).

#### 3.6.4 Studies with two or more treatment groups

Procedures described in the Cochrane Handbook for Systematic Reviews of Interventions will be followed for trials with more than two intervention or comparison arms to avoid double counting of study participants in meta‐analysis (Higgins & Green 2011). Specifically, where possible, active intervention arms will be combined and compared against usual care or control conditions. If this is not possible, a single pair of intervention – control conditions will be selected for comparison. The selection of such a comparison will be undertaken by review pairs who will be blind to results describing intervention effects.

#### 3.6.5 Dealing with missing data

Authors of included trials will be contacted to provide additional information if any outcome data is unclear or missing. Any instances of potentially selective or incomplete reporting of outcome data will be noted in the risk of bias table. Meta‐analysis will be performed using an intention‐to‐treat principle.

The potential impact of missing data on pooled estimates of intervention effects will be examined as part of sensitivity analysis through removal of trials considered at high risk of bias due to attrition in pooled estimates of intervention effects.

#### 3.6.6 Assessment of heterogeneity

For each outcome, we will explore heterogeneity by preparing box plots, and forest plots. We will also quantify the I^2^ statistic that represents the percentage of variability between studies due to heterogeneity (Higgins & Green, 2011). Chi‐squared tests will be used to test the heterogeneity (Higgins & Green, 2011).

#### 3.6.7 Assessment of reporting biases

The comprehensive search strategy of this review will help reduce the risk of reporting bias. Assessment of reporting bias will also be conducted via visual inspection of funnel plots of included studies.

### 3.7 Data synthesis

Meta‐analysis will be performed using a random effects model where two or more trials with suitable data can be identified. This model will allow to estimate the pooled effect size and its 95% confidence interval for each outcome when possible.

Data from randomised and non‐randomised trial designs will not be pooled. Similarly, we will not pool data from non‐randomised studies of different study designs.

Given the variety of possible intervention strategies and outcome measures, we anticipate that a meta‐analysis of included trials will not be possible for the primary review outcome. In this instance, we will describe and synthesise trial findings narratively and/or use descriptive measures (median effect size and range) to describe the effects of trials as conducted in previous reviews (Ivers, 2012).

#### 3.7.1 Summary of findings

A ‘summary of findings’ table presenting the key findings of the review will be included. The table will be generated based on recommendations in the Cochrane Handbook, and include

i) a list of all primary and secondary outcomes of the review,
ii) a measure of absolute and/or relative magnitude of intervention effect (if meta‐analysis is performed)
iii) the number of participants and studies addressing each outcome,
iv) a grade of the overall quality of the body of evidence for each outcome, and
v) any pertinent comments to assist interpretation.

It will provide key information concerning the quality of evidence, the magnitude of effect of the interventions examined, and the sum of available data on the main outcomes.

A PRISMA flow chart and a table of ‘Characteristics of excluded studies’ will also be included.

#### 3.7.2 Subgroup analysis and assessment of heterogeneity

Consistent with recommendations from the Cochrane Handbook, if I^2^>75%, we will split included trials into sub‐groups based on study design, population and intervention characteristics to explain homogeneity. Heterogeneity will also be visually explored using forest and box plots.

Given that a small number of eligible studies are expected for this review, we do not anticipate conducting moderator analyses beyond differences in study design, and it is highly unlikely that we will have a sufficient number of studies to conduct a meta‐regression. As such, we will group studies according to study design.

Specifically, using RevMan we will group studies by whether they are RCTs or non‐RCTs, estimate variance components for within‐studies groups (use fixed effect models if variance components the same, random effects models if different) and test whether the mean effect size from the RCT‐only group differs from the mean effect size from the non‐RCTs group.

#### 3.7.3 Sensitivity analysis

Sensitivity analysis will be performed by removing studies with a high risk of bias. If visual inspection of the forest plots identifies outliers, that is, where the confidence intervals of a trial do not overlap with other included studies, the authors of the trial will be contacted to confirm the trial data and the sensitivity analyses performed with the trial removed to assess any impact on pooled estimates of effect.

#### 3.7.3 Reaching Conclusions

Conclusions derived from the synthesis will be based on findings included in studies only. The conclusions will include a section with implications for both practice and research and describe limitations as well as remaining uncertainties, thereby identifying areas for further examination.

**Table 1:** Scaling strategies applied in included studies

**Study**	**Implementation Strategy/ies tested**
**Author Year**	Program adaptation; task‐shifting
**Author Year**	Train the trainer; fidelity monitoring
**Author Year**	
**Author Year**	

**Table 2:** Outcomes and results of included studies

**Study**	**Outcome**	**Results**
*Author Year*	Adoption	
*Author Year*	Sustainability	
*Author Year*	Costs	

## 6. Review authors

Lead review author:

**Table:** 

Name:	Luke Wolfenden
Title:	Associate Professor
Affiliation:	Hunter New England Population Health
Address:	Locked Bag 10
City, State, Province or County:	Wallsend, New South Wales
Postal Code:	2298
Country:	Australia
Phone:	+61 2 49246567
Email:	luke.wolfenden@hnehealth.nsw.gov.au
**Co‐author(s):**	
Name:	Bianca Albers
Title:	Director
Affiliation:	Centre for Evidence and Implementation ‐ CEI Global UK Ltd
Address:	Suite 1, 3^rd^ Floor, 11‐12 St James's Square
City, State, Province or County:	London
Postal Code:	SW1Y 4LB
Country:	United Kingdom
Phone:	+49 175 109 8379
Email:	bianca.albers@ceiglobal.org
Name: Aron Shlonsky	Aron Shlonsky
Title:	Professor and Head of Department (Social Work)
Affiliation:	Monash University School of Primary and Allied Health Care
Address:	Department of Social Work Building C, Level 4, 900 Dandenong Road
City, State, Province or County:	Caufield East, VIC
Postal Code:	3145
Country:	Australia
Phone:	+61 3 99026011
Email:	aron.shlonsky@monash.edu

## 7. Roles and responsibilities

### 7.1 Content

All three co‐authors will contribute equally with content expertise to this review.

*Dr Luke Wolfenden* is currently co‐authoring systematic reviews registered with the Cochrane Collaboration that focus on the role of implementation strategies in enhancing the implementation of public health interventions (Wolfenden, 2015; Williams et al., 2015). He is also a member of the editorial board of the international journal Implementation Science.

*Bianca Albers* is a practice expert in implementation, who has worked for more than ten years with implementing evidence‐based practices, programs and policies in child, youth and family services. She heads several international professional networks for implementation practitioners and scientists and is the lead developer of the first professional certificate in Implementation Science provided through the University of Melbourne's Department of Social Work.

*Aron Shlonsky* has worked with the Campbell Collaboration for many years, first as co‐chair of the Social Welfare Coordinating group and currently as editor of the Knowledge Translation and Implementation Coordinating Group. Aron has substantial experience in evidence synthesis and services on the Editorial board of Systematic Reviews and Cochrane Child Health. He has also coordinated the professional certificate in Implementation Science with Bianca Albers.

### 7.2 Systematic review methods & statistical analysis

Dr. Wolfenden and professor Shlonsky will provide methodical and statistical expertise to the review team.

Dr *Luke Wolfenden* is a conjoint associate professor at the University of Newcastle and an associate at Hunter New England Population Health, a provider of health services in New South Wales. Dr. Wolfenden worked as an associate training lecturer in systematic reviews at the UK Cochrane Centre, and he has authored and co‐authored several published systematic reviews. Dr. Wolfenden was awarded a prestigious National Health and Medical Research Council (NHMRC) Career Development Fellowship.

Professor *Aron Shlonsky* is a Cochrane/Campbell systematic review author, is the current editor of Campbell's KTI group, and conducts large‐scale data analytics in the child welfare field. Until recently, he was Professor of Evidence Informed Practice at the Department of Social Work at the University of Melbourne School of Health Sciences; the Director of the Victoria Child Welfare Decision‐Making Project; and the Director of the Centre for Applied Research on Effective Services at the University of Melbourne. Professor Shlonsky now holds the position of Head of Department at Monash University's Department of Social Work. Shlonsky is known internationally for his work in risk assessment for child maltreatment and domestic violence, child welfare practice and policy, data analytics and the use of evidence to inform practice and policy.

### 7.3 Information retrieval

Two highly experienced librarians will conduct literature searches for this review.

Debra *Frances Booth* is leading the information retrieval team. She is a faculty librarian at the Faculty of Health and Medicine at The University of Newcastle, where she provides leadership and advice on the development of strategic information policy and services required to support the University Library's learning. Ms. Booth is an experienced academic librarian with expertise across health, medicine and social science databases. She has designed and executed search strategies and managed citations for approximately 90 systematic reviews for academics at the University of Newcastle, three of which have been conducted for the Cochrane Collaboration.

*Tania Celeste* is a librarian and a passionate information retrieval specialist working for the University of Melbourne (UoM) as a liaison librarian specialized in health‐related topics. Tania will contribute with her specialist expertise on systematic searches in the production of this systematic review, and support the lead librarian, Ms. Booth.

### 7.4 Process management

Bianca Albers will head the daily management of the production of this systematic review.

She will manage processes necessary to coordinate and progress the work with this systematic review and be involved in all phases of the review work, including literature screening, data collection and writing. Bianca has extensive experience in processes of research synthesis and translation, has co‐authored several rapid and scoping reviews and co‐tutors a course in scoping reviews provided to social work students at the University of Melbourne.

Furthermore, two research assistants recruited through the University of Melbourne and University of Newcastle will support the production of this systematic review. Both have substantial experience with conducting, coordinating and managing literature searches and will be supervised by assistant professor Luke Wolfenden.

### 7.5 Advisory Panel

An advisory panel is linked to this systematic review to provide input on especially content‐related questions linked to the topics ‘scaling’, ‘implementation measurement’ and ‘implementation strategies’. Members of this advisory panel are:

1. **Byron J. Powell**, assistant professor for health policy and management at the Gillings School of Global Public Health at the University of North Carolina, US. Dr Powell's research focuses on efforts to improve the quality of behavioural health and social services provided in community settings. Specifically, he is working to develop a better understanding of the types of strategies that can be used to implement effective services, and the organizational and systemic factors that can facilitate or impede implementation and quality improvement.
2. **Greg Aarons**, professor at the Department of Psychiatry at the University of California San Diego, US. Professor Aarons is also the Director of the Child and Adolescent Services Research Center (CASRC) and Co‐Director of the Center for Organizational Research on Implementation and Leadership (CORIL). His research focuses on identifying and improving system, organizational, and individual factors that impact successful implementation and sustainment of evidence‐based practices and quality of care in health care and public sector practice settings.
3. **Cara Lewis**, Assistant Professor, Department of Psychological & Brain Sciences, Indiana University, US. Dr Lewis' research focuses on factors and processes related to the successful dissemination and implementation of evidence‐based programs in the community. She serves as principal investigator of a research project focused on comparing standardized versus tailored approaches to implementing measurement‐based care. Dr Lewis is President of the Society for Implementation Research Collaboration (SIRC) and co‐director of the SIRC conference series. Through SIRC, she serves as principal investigator on an additional research project focused on developing measures and methods to advance implementation science.
4. **Nick Sevdalis**, Professor of Implementation Science and Patient Safety, King's College London, UK. Nick Sevdalis is also the director of the Centre for Implementation Science and leads the Collaboration for Leadership in Applied Health Research and Care (CLAHRC) South London implementation science research team. The team supports the work of the CLAHRC in its eight specialty areas (alcohol, diabetes, infection, maternity and women's health, palliative and end of life psychosis, public health and stroke) and seeks to develop the discipline of implementation science.
5. **Sharon Licqurish,** Research Fellow, Department of General Practice and Primary Care Academic Centre, University of Melbourne. Sharon is a qualitative researcher who has successfully applied rigorous qualitative methodology and theoretical perspectives to develop substantive theory about social action. Her area of expertise is Cancer research in Primary Care. She is working on a program of research exploring cancer beliefs in migrant populations and developing community‐based interventions to facilitate timely diagnosis in culturally and linguistically diverse populations. Her research experience is broad and includes cancer in primary care, work with culturally and linguistically diverse communities, women's' health and midwifery practice. Sharon holds a strong interest in Implementation Science methodology, translation of research findings into practice and integrated health care.
6. **Kathleen Conte**, Research Fellow, Australian Prevention Partnership Centre, Menzies Centre for Health Policy, University of Sydney, Australia. Dr Conte's research interests are in developing health prevention systems through intersecting policy, research and practice.
7. **France Légaré,** Tier 1 Canada Research Chair in Shared Decision Making and Knowledge Translation, Department of Family Medicine and Emergency Medicine, Faculty of Medicine, Laval University, Québec, Canada. France Légaré practices family medicine in Quebec and is a full professor in the Department of Family Medicine and Emergency Medicine at Université Laval, Quebec. In 2005, she obtained her PhD in Population Health from the University of Ottawa. From June 2006 to May 2016, Dr. Légaré has held the title of Tier 2 Canada Research Chair in Implementation of Shared Decision Making in Primary Care. She has also been the Canadian Cochrane Network Site representative at Université Laval (the CHUQ Research Centre) from 1999 to 2013; she has launched in 2013 Cochrane Canada Francophone and now serves as its co‐director. She is working closely with Herve Zomahoun PhD scientific coordinator of Health and Social Services Systems, Knowledge Translation and Implementation component of the Québec CIHR SPOR SUPPORT Unit. Herve Zomahoun has a PhD in pharmacoepidemiology and teaches systematic reviews methods in the Master and doctoral programs of epidemiology at Université Laval. She also works closely with Ali Ben Charif PhD is a postdoctoral Fellow, Health and Social Services Systems, Knowledge Translation and Implementation component of the Québec CIHR SPOR SUPPORT Unit. Dr Ben Charif's research interest is in scaling up evidence‐based practices in community‐based primary health care. As part of this review, both Dr Zomahoun and Dr Charif will contribute to the Advisory Panel discussions.

## 8. Sources of support

This systematic review receives financial support (38,859 USD) from the Campbell Collaboration / the American Institutes for Research (AIR).

Members of the advisory committee provide their time as in‐kind contribution to the production of this systematic review.

## 9. Declarations of interest

The authors declare to have no competing interests.

## 10. Preliminary timeframe

- Submission of a draft protocol: 1.7.2017
- Submission of a draft review: 1.2.2019
- Submission of final review: 1.10.2019

## 11. Plans for updating the review

The review, once completed, will be updated every 3^rd^ year.

Responsible for the update is the team of three co‐authors.

## AUTHOR DECLARATION

### Authors' responsibilities

By completing this form, you accept responsibility for preparing, maintaining and updating the review in accordance with Campbell Collaboration policy. The Campbell Collaboration will provide as much support as possible to assist with the preparation of the review.

A draft review must be submitted to the relevant Coordinating Group within two years of protocol publication. If drafts are not submitted before the agreed deadlines, or if we are unable to contact you for an extended period, the relevant Coordinating Group has the right to de‐register the title or transfer the title to alternative authors. The Coordinating Group also has the right to de‐register or transfer the title if it does not meet the standards of the Coordinating Group and/or the Campbell Collaboration.

You accept responsibility for maintaining the review in light of new evidence, comments and criticisms, and other developments, and updating the review at least once every five years, or, if requested, transferring responsibility for maintaining the review to others as agreed with the Coordinating Group.

### Publication in the Campbell Library

The support of the Coordinating Group in preparing your review is conditional upon your agreement to publish the protocol, finished review, and subsequent updates in the Campbell Library. The Campbell Collaboration places no restrictions on publication of the findings of a Campbell systematic review in a more abbreviated form as a journal article either before or after the publication of the monograph version in Campbell Systematic Reviews. Some journals, however, have restrictions that preclude publication of findings that have been, or will be, reported elsewhere and authors considering publication in such a journal should be aware of possible conflict with publication of the monograph version in Campbell Systematic Reviews. Publication in a journal after publication or in press status in Campbell Systematic Reviews should acknowledge the Campbell version and include a citation to it. Note that systematic reviews published in Campbell Systematic Reviews and co‐registered with the Cochrane Collaboration may have additional requirements or restrictions for co‐publication. Review authors accept responsibility for meeting any co‐publication requirements.

**I understand the commitment required to undertake a Campbell review, and agree to publish in the Campbell Library. Signed on behalf of the authors**:

**Table:** 

**Form completed by: Bianca Albers**	**Date: 1 August 2018**

## Footnotes

1
http://www.ucl.ac.uk/gacd/research/implementation‐science

## Appendix

**Table:**

**#**	**Searches**	**Results**
1	(“scal* up” or “scal* out” or scaling or scalability or “bringing to scale” or “bringing up to scale” or “at scale” or “at capacity”).mp.	‘Scaling’ terms
2	sustainab*.mp.	
3	1 or 2	
4	implement*.mp.	‘Implementation’ terms
5	dissemin*.mp.	
6	adopt*.mp.	
7	diffus*.mp.	
8	quality improvement*.mp.	
9	transform*.mp.	
10	translat*.mp.	
11	transfer*.mp.	
12	uptake*.mp.	
13	incorporat*.mp.	
14	integrat*.mp.	
15	4 or 5 or 6 or 7 or 8 or 9 or 10 or 11 or 12 or 13 or 14	
16	((polic* or practice* or program* or innovation* or guideline*) and (performance or feedback or effective or evidence or evidence‐informed or ‐evidence‐based)).mp.	‘Intervention’ terms
17	randomized controlled trial/	‘Study design’ terms
18	controlled clinical trial/	
19	clinical trials as topic/	
20	trial.tw.	
21	qua?irandomi*.tw.	
22	Controlled Before‐After Studies/	
23	Interrupted Time Series Analysis/	
24	time series.tw.	
25	regression discontinuity.mp.	
26	before after.tw.	
27	step* wedge.mp.	
28	(difference in difference or difference of difference).mp.	
29	propensity score/	
30	propensity score*.tw.	
31	matching design*.mp.	
32	(Control group* or comparison group* or pre‐post or pre post or panel stud*).mp.	
33	randomi*.tw.	
34	random* allocate*.mp.	
35	17 or 18 or 19 or 20 or 21 or 22 or 23 or 24 or 25 or 26 or 27 or 28 or 29 or 30 or 31 or 32 or 33 or 34	
**36**	**3 and 15 and 16 and 35**	**Final strategy**
